# Toll-Like Receptor Signalling Is Not Involved in Platelet Response to *Streptococcus pneumoniae In Vitro* or *In Vivo*

**DOI:** 10.1371/journal.pone.0156977

**Published:** 2016-06-02

**Authors:** Sacha F. de Stoppelaar, Theodora A. M. Claushuis, Marianne C. L. Schaap, Baidong Hou, Tom van der Poll, Rienk Nieuwland, Cornelis van ‘t Veer

**Affiliations:** 1 Center for Infection and Immunity Amsterdam (CINIMA), University of Amsterdam, Amsterdam, the Netherlands; 2 Center for Experimental and Molecular Medicine (CEMM), University of Amsterdam, Amsterdam, the Netherlands; 3 Laboratory for Experimental and Clinical Chemistry (LEKC), University of Amsterdam, Amsterdam, the Netherlands; 4 Key Laboratory of Infection and Immunity, Institute of Biophysics, Chaoyang District, Beijing, China; 5 Division of Infectious Diseases, University of Amsterdam, Amsterdam, the Netherlands; University of Leuven, BELGIUM

## Abstract

*Streptococcus (S*.*) pneumoniae* strains vary considerably in their ability to cause invasive disease in humans, which is at least in part determined by the capsular serotype. Platelets have been implicated as sentinel cells in the circulation for host defence. One of their utensils for this function is the expression of Toll-like receptors (TLRs). We here aimed to investigate platelet response to *S*. *pneumoniae* and a role for TLRs herein. Platelets were stimulated using four serotypes of *S*. *pneumonia* including an unencapsulated mutant strain. *In vitro* aggregation and flow cytometry assays were performed using blood of healthy volunteers, or blood of TLR knock out and WT mice. For *in vivo* pneumonia experiments, platelet specific *Myd88* knockout (Plt-*Myd88*^-/-^) mice were used. We found that platelet aggregation was induced by unencapsulated *S*. *pneumoniae* only. Whole blood incubation with all *S*. *pneumoniae* serotypes tested resulted in platelet degranulation and platelet-leukocyte complex formation. Platelet activation was TLR independent, as responses were not inhibited by TLR blocking antibodies, not induced by TLR agonists and were equally induced in wild-type and *Tlr2*^*-/-*^, *Tlr4*^*-/-*^, *Tlr2/4*^*-/-*^, *Tlr9*^*-/-*^ and *Myd88*^*-/-*^ blood. Plt-*Myd88*^*-/-*^ and control mice displayed no differences in bacterial clearance or immune response to pneumonia by unencapsulated *S*. *pneumoniae*. In conclusion, *S*. *pneumoniae* activates platelets through a TLR-independent mechanism that is impeded by the bacterial capsule. Additionally, platelet MyD88-dependent TLR signalling is not involved in host defence to unencapsulated *S*. *pneumoniae in vivo*.

## Introduction

*Streptococcus (S*.*) pneumoniae* is a frequent inhabitant of the upper airways in healthy individuals, but also the most common cause of community-acquired pneumonia and a main cause of sepsis [1, 2]. Sepsis is a life-threatening condition, where the host response to infection is injurious to tissues and organs [3]. During sepsis, activation of the coagulation cascade, together with endothelial damage, leads to platelet activation. Platelets can additionally be activated by pathogens and components thereof during bacterial dissemination [4–7]. Sepsis patients indeed show an increase in platelet activation markers [8, 9] and a decrease in platelet counts [10, 11], and the extent of these responses is associated with mortality.

Platelets are widely renowned for their role in haemostasis. More recently, platelets have been implicated as major players in host defence [4, 6]. The platelet releasate contains a number of pro-inflammatory proteins and antimicrobial peptides [6, 7]. Platelet activation and P-selectin expression lead to platelet-neutrophil interaction, which recruit neutrophils to an inflammatory site [6, 12] and stimulate the release of neutrophil extracellular traps [6, 13]. Platelet depletion *in vivo* leads to enhanced bacterial growth and increased mortality during murine *S*. *pneumoniae* [14] and *Klebsiella pneumoniae* [15] induced pneumosepsis.

Platelets express several immune related receptors such as Toll-like receptor (TLR) 1, 2, 4–7 and 9, receptors for Fc domain of IgG FcγRII and FcɛRI, complement receptors, and cyto- and chemokine receptors [16]; additionally platelet protease activated receptor (PAR)1 (human platelets), PAR3 (mouse platelets), PAR4 (both species), glycoprotein (GP)IV, GPIIbIIIa and GPIbα can play a role in inflammatory reactions [16]. TLRs are a family of pattern recognition receptors that are critical for microbial surveillance and regulation of inflammatory and immune responses [17]. Functional roles for some platelet TLRs have been described [13, 18, 19], indicating that they are not residual receptors conserved from their bone marrow precursors. However, several reported functions of platelet TLR’s have been questioned as discussed in detail by Kerrigan and Cox [20].

Considering the important role for platelets in host defence to *S*. *pneumoniae* [14], we here aimed to investigate whether and how *S*. *pneumoniae* can directly activate platelets. For this, we measured *S*. *pneumoniae* induced platelet activation in a variety of assays in human and mouse blood, investigated a possible role for TLR signalling herein, and performed *in vivo* pneumonia experiments with platelet specific MyD88 depleted (Plt-*Myd88*^*-/-*^) mice to determine the potential role of TLR mediated MyD88 signaling in platelets during *S*. *pneumoniae* induced pneumosepsis. We found that *S*. *pneumoniae* activates platelets through a TLR-independent mechanism that is impeded by the bacterial capsule and that platelet MyD88-dependent TLR signalling is not involved in host defence to unencapsulated *S*. *pneumoniae in vivo*.

## Materials and Methods

### Aggregation assay

Optical platelet aggregation was assayed with human platelet rich plasma on the aggregometer PAP-8E (Bio/data corporation, Horsham, PA) at 900 rpm and 37°C according to manufacturer’s instruction. Citrate-anticoagulated whole blood was collected from healthy volunteers. Platelet-rich plasma (PRP) was obtained by centrifugation at 180 g for 15 minutes at room temperature (RT). PRP was recentrifuged at 1500 g for 10 minutes to obtain platelet-poor plasma (PPP). Stimuli used were: *S*. *pneumoniae* serotype 2 (D39), *S*. *pneumoniae* serotype 3 (6303), *S*. *pneumoniae* serotype 4 (TIGR4), unencapsulated *S*. *pneumoniae* D39 (ΔcpsD39 [21]), lipoteichoic acid (LTA; 5 μg/mL; *S*. *aureus*, Invivogen, San Diego, CA), Pam3CSK4 (5 μg/mL; Invivogen), lipopolysaccharide (LPS; ultrapure 5 μg/mL; *E*. *coli*, Invivogen) and recombinant *S*. *pneumoniae* serotype 2 capsule (rCPS2, 10 μg/mL; ATCC, Manassas, VA). Maximum platelet aggregation was determined in the presence of thrombin receptor activating peptide (TRAP, 15 μM; Sigma-Aldrich, St. Louis, MO). Indicated inhibitors were added 15 minutes prior to stimulation: Abciximab (ABC, glycoprotein IIbIIIa inhibitor Reopro, 10 μg/mL; Eli Lilly, Houten, the Netherlands), prostaglandin E1 (PGE1, 100 nM; Sigma-Aldrich), anti (α)-TLR2 (5 μg/mL, clone T2.5, blocking antibody; HBT, Uden, the Netherlands), α-TLR4 (5 μg/mL, clone 18H10, blocking antibody; HBT) and α-FcγRII (25 μg/mL, clone AT10, blocking antibody; Abcam, Cambridge, UK). To evaluate platelet priming, PRP was stimulated for 5 minutes under stirring conditions at 37°C, before adding subthreshold concentration of TRAP. For each experiment, the TRAP concentration inducing the minimal measurable aggregation (hereby defined threshold concentration) was determined; usually 234 nM. Peripheral blood mononuclear cells (PBMCs) were isolated using Polymorphprep^™^ (Frensenius Kabi, Oslo, Norway) according to manufacturer’s instructions. Recordings were stopped after 10 or 15 minutes. The medical ethical committee of the Academic Medical Center in Amsterdam gave ethical approval for the conduction of the study (no. NL 34294.018.10) and written informed consent was obtained from all healthy controls.

### Validation of anti-TLR2 antibodies

Anti-TLR2 antibodies T2.5, TLR2.45, TL2.1 (kindly provided by HBT, Uden, The Netherlands) were tested for their ability to inhibit TLR2 function by 30 minutes pre-incubation of the antibodies in heparinized human whole blood and stimulation with 300ng/mL of TLR2 ligand PAM3CSK4 added by an equal of the ligand in RPMI1640 medium supplemented with 0.1% human albumin and overnight incubation at 37°C and determination of released TNFα using ELISA (BD Biosciences Pharmingen (San Diego, CA).

Further testing of the inhibitory anti-TLR2 antibody T2.5 to inhibit responses by pure TLR2 ligands and *S*. *pneumoniae* responses was performed on HEK293 cells, stably transfected with TLR2 and CD14 [22, 23]. These cells were stimulated overnight with LTA (5 μg/mL), Pam3CSK4 (300 ng/mL) or 10^6^ CFU ΔcpsD39, after a 30 minutes pre-incubation with 5 μg/mL α-TLR2 or medium control. Following 16 hours of stimulation at 37°C, supernatant was collected and IL-8 was determined using ELISA (R&D Systems, Abingdon, UK).

### Flow cytometry

45 μL of citrated human or mouse whole blood was stimulated with 5 x 10^7^ viable CFUs *S*. *pneumoniae* D39, ΔcpsD39, 6303 and TIGR4 in 45 μL PBS. Maximum platelet activation was determined in the presence of 15 μM TRAP. Where indicated, inhibitors were added 15 minutes prior to stimulation. Inhibitors were diluted in HEPES buffer (137 mM NaCl, 2.7 mM KCl, 1 mM MgCl_2_, 20 mM HEPES, 3.3 mM NaH_2_PO_4_, 1 g/l bovine serum albumin, 5.6 mM D-glucose, pH 7.4) and added in 5 μL to a final concentration of 5 μg/mL for α-TLR2, 10 μg/mL for Abciximab and 25 μg/mL for α-FcγRII AT10. Following 30 minutes incubation at RT, 5 μL stimulated blood was added to a mixture of antibodies in HEPES buffer, i.e., anti-CD61-APC (Dako, Heverlee, Belgium), anti-CD62p-PE (Beckman Coulter, Woerden, the Netherlands), anti-CD63-FITC (Beckman Coulter), anti-CD45-APC (BD biosciences, San Jose, CA), anti-CD14-FITC (BD biosciences) or isotype controls for human studies and anti-CD61-APC (BioLegend, San Diego, CA), anti-CD62p-FITC (Clone RB40.34, BD biosciences) and isotype controls for mice and incubated at RT for 30 minutes. For platelet analysis, samples were fixed by addition of 2.5 mL 0.3% paraformaldehyde-containing HEPES-buffer. For platelet-leukocyte complex analysis, samples were fixed by addition of 0.5 mL 0.3% paraformaldehyde-containing HEPES-buffer and erythrocytes were subsequently lysed by addition of 1.8 mL aquadest following centrifugation for 10 minutes at 400g, after which pellets were resuspended in 200 μL HEPES buffer. Toll like receptor 2 (Clone T2.5, Ebioscience) and Toll like receptor 4 (clone HTA125, eBioscience) were measured on platelets fixated with 1% paraformaldehyde. Results are representative of 2 independent experiments with 2 different donors.

Flow cytometry was performed on a Calibur flow cytometer (Becton Dickinson, Franklin Lakes, NJ). Data were analysed using FlowJo (V10.0.4).

### Animals

Specific pathogen-free C57Bl/6 mice were purchased from Harlan Sprague-Dawley (Horst, the Netherlands). *Tlr2*^*-/-*^, *Tlr4*^*-/-*^, *Tlr9*^*-/-*^ and *MyD88*^*-/-*^ mice were generously provided by prof. Shizuo Akira (Research Institute for Microbial Disease, Osaka, Japan) [24–26]. *Tlr2/4*^*-/-*^ double knock out mice were crossed from *Tlr2*^*-/-*^ and *Tlr4*^*-/-*^ as described [27]. MyD88 floxed mice (*Myd88*^*lox/lox*^) were kindly provided by prof. Anthony DeFranco [28]. Platelet specific MyD88 knock out (Plt-*Myd88*^*-/-*^) mice were generated by crossing these with mice expressing Cre recombinase under the platelet factor 4 (PF4) promoter (The Jackson Laboratory, Bar Harbor, Maine); littermates not expressing Cre were used as controls.

Mice were housed in a the animal facility of the Academic Medical Center with a 12 hour day-night rhythm, food and water ad libitum, temperature and moisture control, and daily checks. Upon arrival in the facility, mice were acclimatized for at least 7 days before use in experiments. Mice were euthanized by cervical dislocation after anesthesia with (0.12mg/g body weight) ketamine and (0.3ug/g body weight) dexmedetomidine intraperitoneally. Mice were monitored minimally once daily during experiments. Human endpoint for infection experiments was if mice were segregated from the others and unable to lift themselves from supine position. No mice reached the human endpoint before the experimental endpoint. The Institutional Animal Care and Use Committee of the Academic Medical Center approved all experiments (Permit Number DIX21BR and DIX101643).

### Experimental study design

Pneumonia was induced by intranasal inoculation with ΔcpsD39 (2 x 10^7^ colony forming units (CFU) in 50 μL isotonic saline) using previously described methods [21, 29]. Mice were euthanized 16 hours after induction of pneumonia (N = 7/9 mice per group). Bacterial quantification and storage of organs were performed as described [21, 29], platelet counts and activation (by expression of P-selectin as described above) were determined in citrated whole blood by flow cytometry. Mouse tumour necrosis factor (TNF-)α, interleukin (IL-)6, IL-1β, keratinocyte chemoattractant (KC), PF4, soluble (s)P-selectin, E-selectin (R&D Systems) and thrombin-antithrombin complexes (TATc; Bio-connect, Huissen, the Netherlands) were measured by ELISA. Four-micrometer sections of the left lung lobe, spleen and liver were stained with hematoxylin and eosin (H&E). To make sure sections were representative of the entire lung, sections were first carefully cut into the middle part of the fixated lung and assessed by a blinded pathologist before scoring. Slides were coded and scored by a pathologist blinded for group identity for the following parameters: infiltrative surface (expressed as the percentage of total lung surface), bleeding, infiltration, interstitial inflammation, endothelialitis, bronchitis, oedema, pleuritis and presence of thrombi. All parameters were rated separately from 0 (condition absent) to 4 (most severe condition). The total histopathological score was expressed as the sum of the scores of the individual parameters.

### Statistical analysis

All analyses were done using GraphPad Prism version 5.01 (GraphPad Software, San Diego, CA). Comparisons between groups (8 mice per group) were tested using the Mann-Whitney U test as data was non-parametric.” P-values < 0.05 were considered statistically significant.

## Results

### Unencapsulated, but not encapsulated, *S*. *pneumoniae* induces platelet aggregation

**Fig 1:**
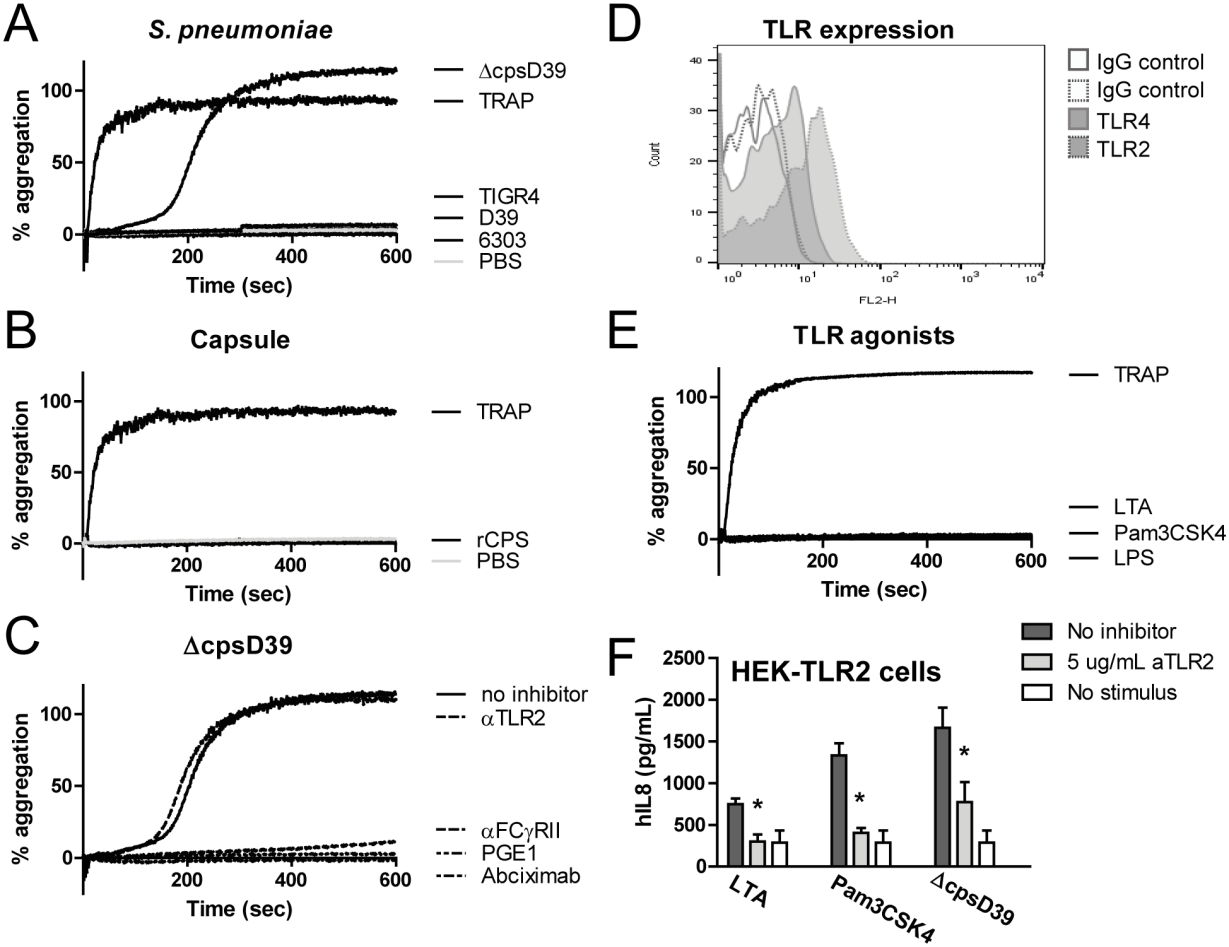
Unencapsulated, but not encapsulated, *S*. *pneumoniae* induces human platelet aggregation. Platelet aggregation was measured by light transmission as a percentage to the transmission through PPP in a stirring cuvette. TRAP was used as a positive control; PBS as a negative control. Aggregation curves are depicted for stimulation with *S*. *pneumoniae* D39, ΔcpsD39, TIGR4 and 6303 (A) and rCPS2 (B). PRP was pre-incubated with α-TLR2, α-FcyRII, PGE1, abciximab or PBS prior to ΔcpsD39 stimulation in (C). Toll like receptor 2 and 4 expression on human platelets are shown in (D). Aggregation curves as a result of TLR agonist stimulation with LTA, Pam3CSK4 or LPS are shown in (E). All aggregation curves are representative of 3 independent experiments using different donors. HEK cells stably transfected with TLR2 and CD14 were pre-incubated with α-TLR2 and stimulated for 16 hours with LTA, Pam3-CSK4 and ΔcpsD39, IL-8 was measured in the supernatant (n = 4) (F). * P<0.05.

Unencapsultated ΔcpsD39 *S*. *pneumonia serotype 2* induced platelet aggregation in human platelet rich plasma consistent with the observations of Keane *et al* [30] that unencapsulated *S*. *pneumoniae* causes platelets to aggregate. The encapsulated D39 serotype 2, *as well as S*. *pneumoniae* serotype, 3 and 4 (6303 and TIGR4 respectively) failed to induce platelet aggregation (Fig 1A). The finding that capsulated S. pneumoniae did not induce aggregation was consistent with the lack of aggregation in the presence of a recombinant preparation of *S*. *pneumoniae* capsule (CPS2) (Fig 1B). Platelet aggregation by ΔcpsD39 was activation dependent, required fibrinogen binding to GPIIbIIIa and FcγRII occupation as it could be inhibited by PGE1, the GPIIbIIIa antagonist Abciximab and the FcγRII antagonist AT10 (Fig 1C). In this respect the platelet aggregation induced by ΔcpsD39 is in perfect agreement with previous reports [30, 31].

Platelets express Toll like receptors 2 and 4, which have been previously described to be functional [19, 30, 32–34]. Using Flow cytometry, we could also detect Toll like receptor 2 and 4 on human platelets (Fig 1D). However, blocking of TLR2 did not affect aggregation (Fig 1C) which is in contrast to the TLR2-dependent *S*. *pneumoniae*-induced platelet activation described by Keane *et al* [30]. Moreover, stimulation with the purified TLR2 agonists Pam3CSK4 and LTA and the TLR4 agonist LPS failed to induce any response even at high concentrations (5 μg/mL; Fig 1E). We confirmed the capacity of the used TLR2 antibody T2.5 to inhibit TLR2 responses in other assays. First we showed that T2.5 is a superior TLR2 blocking antibody compared to other TLR2 antibodies in a whole blood assay (S1 Fig). Additionally, TLR2 activation by *S*. *pneumoniae* ΔcpsD39 is inhibited by T2.5 (Fig 1F). These results indicate that *S*. *pneumonia*e may aggregate platelets in a TLR2 independent manner.

### Prestimulation with *S*. *pneumoniae* fails to modulate platelet aggregation to subthreshold concentrations of TRAP

**Fig 2:**
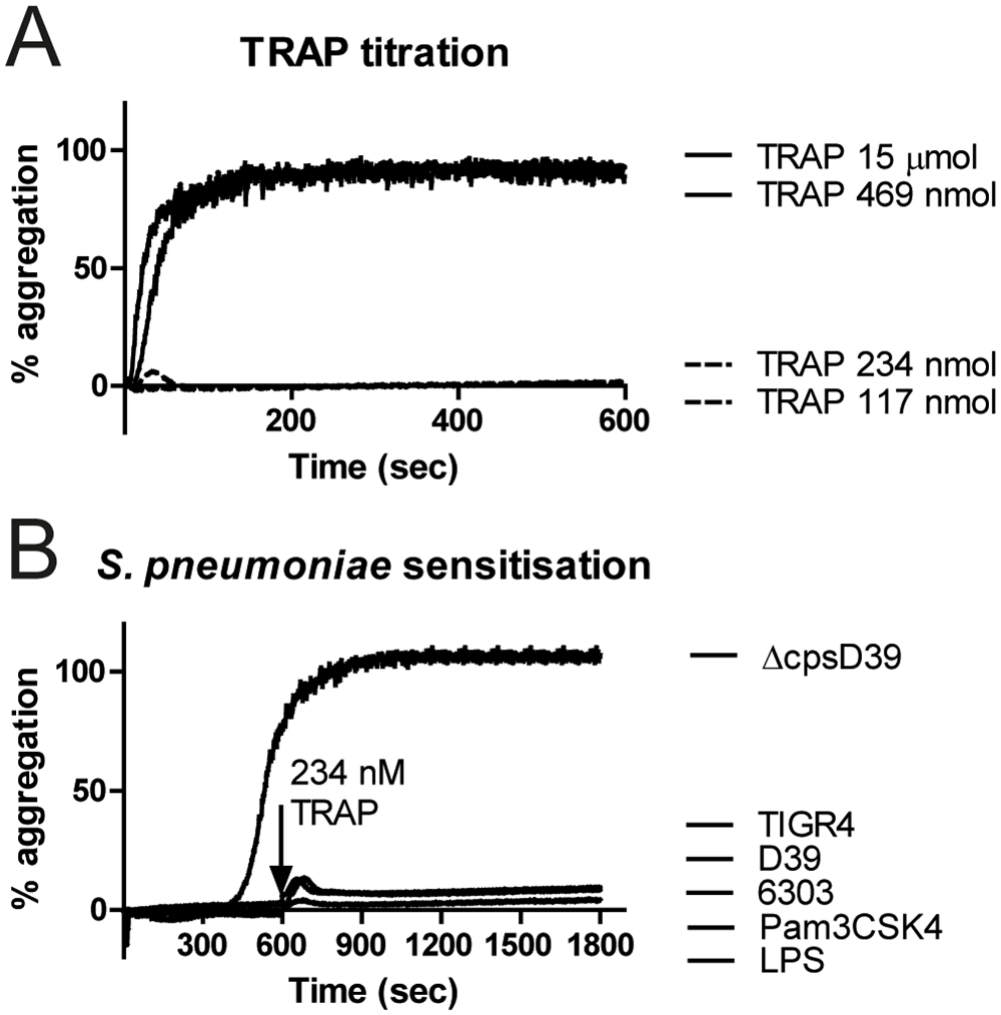
Prestimulation with *S*. *pneumoniae* fails to modulate human platelet aggregation in response to subthreshold concentrations of TRAP. Aggregation curves testing minimal TRAP concentration to induce aggregation are depicted in (A). Prior to stimulation with this subthreshold TRAP concentration, PRP was incubated with *S*. *pneumoniae* D39, ΔcpsD39, TIGR4 and 6303 for 10 minutes in a stirring cuvette (B). Aggregation curves are representative of 3 independent experiments using different donors.

Previous studies have described a role for LPS in platelet ‘priming’, where LPS pretreatment induced platelet hypersensitivity to subthreshold concentrations of classical platelet agonists (24;25). However, we failed to observe any priming effect of pre-incubation of platelets with either *S*. *pneumoniae* or TLR agonists before stimulation with subthreshold concentration TRAP (Fig 2A and 2B). The priming effect of LPS described by Montrucchio (24) was monocyte-dependent; we therefore repeated these experiments in the presence of isolated PBMC’s. Still, no platelet hypersensitivity to subthreshold TRAP was found (S2 Fig).

### S. pneumoniae *D39*, *ΔcpsD39*, *TIGR4 and 6303 induce platelet degranulation*

**Fig 3:**
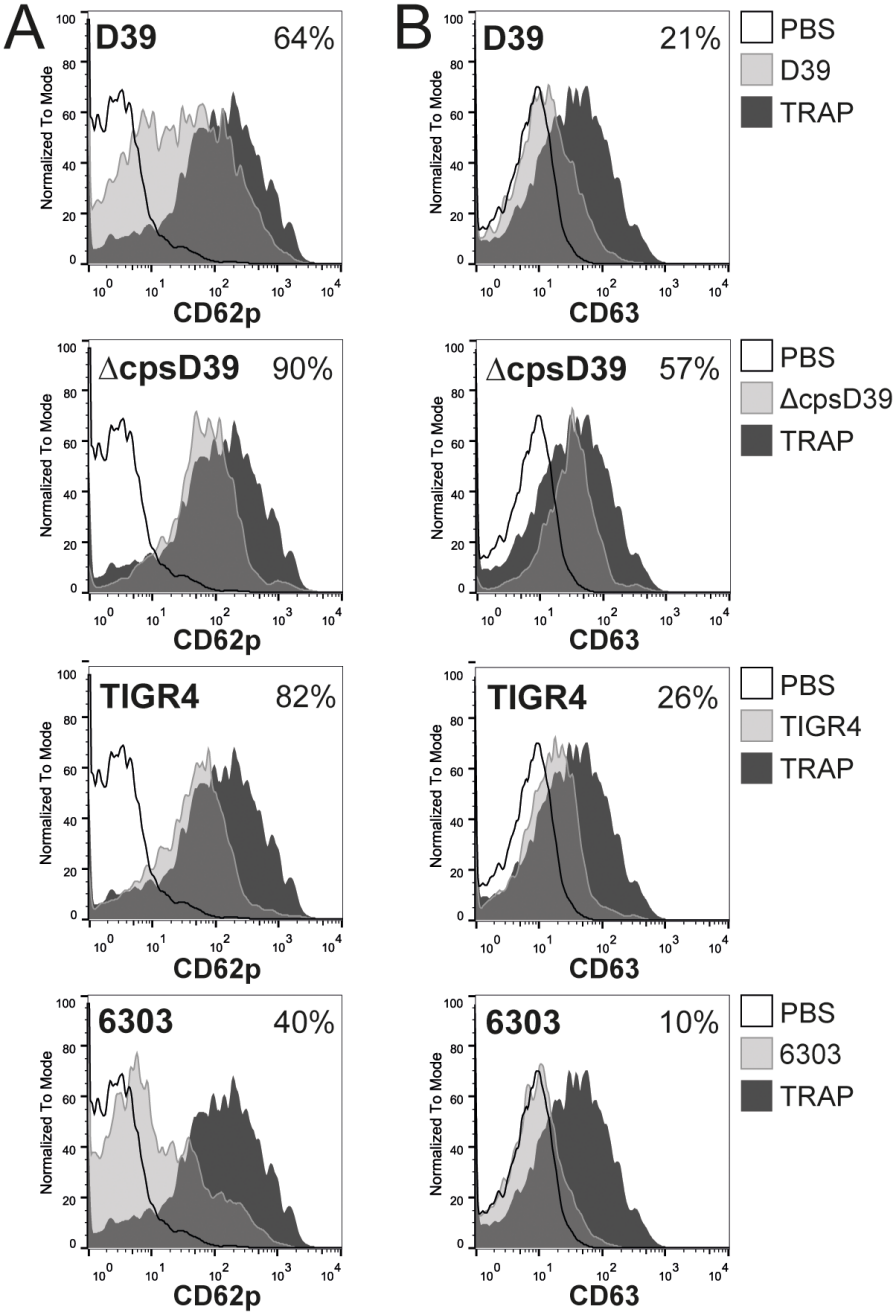
*S*. *pneumoniae* D39, ΔcpsD39, TIGR4 and 6303 all induce human platelet degranulation. Whole blood was stimulated with *S*. *pneumoniae* D39, ΔcpsD39, TIGR4 or 6303. Following 30 minutes of incubation platelets were stained and analysed by flow cytometry for surface expression of CD62p (A) and CD63 (B). Percentages were determined using isotype control antibodies to set the gate. TRAP was used as a positive control and induced CD62p—and CD63 expression on 87% and 56% of platelets respectively; PBS induced CD62p—and CD63 expression on 10% and 2% of platelets. Histograms are representative of 2 independent experiments using different donors.

**Fig 4:**
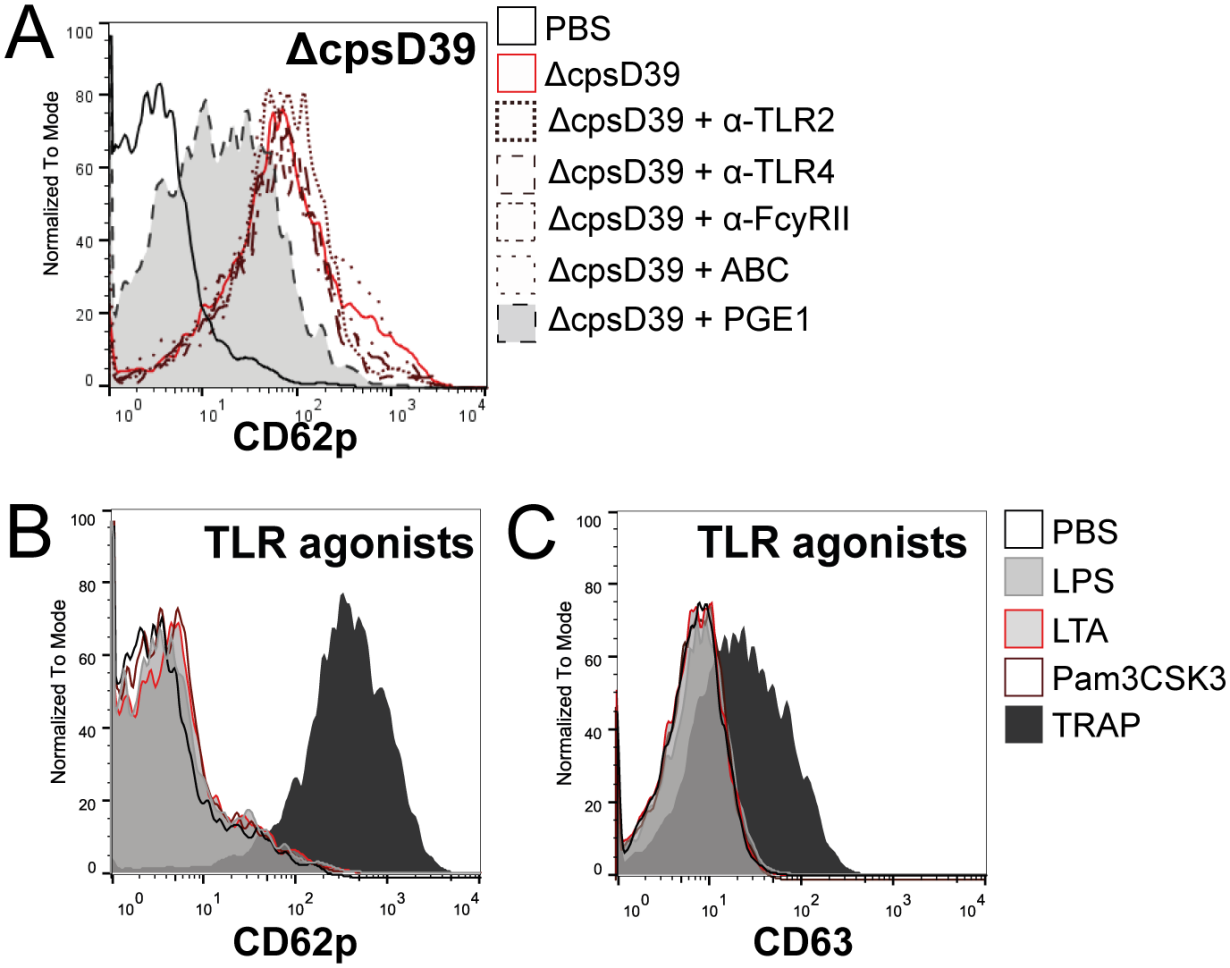
TLR2, 4, FcyRII and GPIIb/IIIa are not involved in *S*. *pneumoniae* induced human platelet degranulation. Following pre-incubation with blocking antibodies to TLR2, TLR4, or FcyRII, or GPIIb/IIIa (Abciximab (ABC)) or with PGE1, whole blood was stimulated with *S*. *pneumoniae* ΔcpsD39. Platelets were stained and analysed by flow cytometry for surface expression of CD62p (A). As an opposite approach, whole blood was incubated with TLR2 and 4 agonists LTA, Pam3CSK4 and LPS and analysed by flow cytometry for surface expression of CD62p (B) or CD63 (C). Histograms are representative of 2 independent experiments using different donors.

Platelet activation by different agonists can induce a variety of responses. We therefore focused on platelet granule release. Alpha granule degranulation was detected by CD62p (P-selectin) surface expression and dense granule release was detected based on surface expression of CD63 [35]. Whole blood stimulation by *S*. *pneumoniae* D39, 6303, TIGR4 and ΔcpsD39 all resulted in platelet CD62p and CD63 exposure, ΔcpsD39 being the most potent activator (Fig 3). *S*. *pneumoniae* did not activate platelets via TLR2 or 4, as pre-incubation with α-TLR2 and α-TLR4 did not inhibit CD62p expression by *S*. *pneumoniae* (Fig 4A). Opposed to aggregation, FcγRII and GPIIbIIIa inhibition did not block CD62p expression by *S*. *pneumoniae* D39 or ΔcpsD39, but PGE1 did (Fig 4A). Platelet surface expression of CD62p or CD63 was not induced by direct TLR agonists LTA, Pam3CSK4 or LPS (Fig 4B and 4C).

### Whole blood *S*. *pneumoniae* incubation results in platelet-leukocyte complex formation

**Fig 5:**
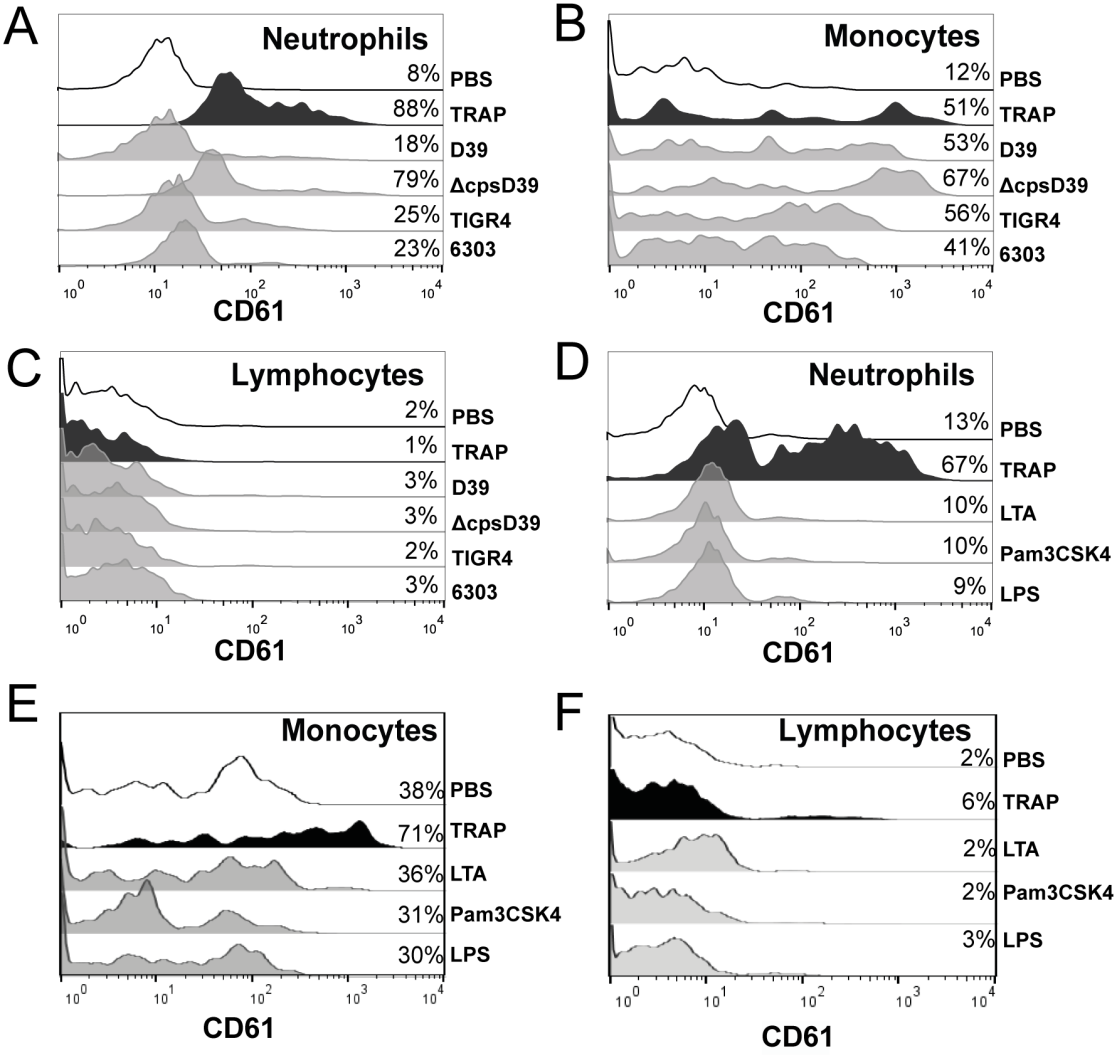
Human whole blood *S*. *pneumoniae* incubation results in platelet-leukocyte complex formation. Whole blood was stimulated with *S*. *pneumoniae* D39, ΔcpsD39, TIGR4 or 6303. Following 30 minutes of incubation leukocytes subsets were stained and analysed for surface expression of CD61. Percentages were determined using isotype control antibodies to set the gate. Neutrophil-CD61 is depicted in (A), monocyte-CD61 in (B) and lymphocyte-CD61 in (C). TRAP was used as a positive control and PBS as a negative control. Histograms are representative of 2 independent experiments using different donors. In a similar fashion, neutrophil-platelet (D) and monocyte-platelet (E) and lymphocyte-platelet (F) complex formation was analysed following stimulation with TLR agonists LTA, Pam3CSK4 and LPS.

To determine whether *S*. *pneumoniae* whole blood stimulation results in formation of platelet-leukocyte complexes, platelet markers CD61 and CD62p were measured on neutrophils, monocytes and lymphocytes (shown for CD61 in Fig 5). All *S*. *pneumoniae* strains tested induced some platelet-neutrophil complexes; ΔcpsD39 being the most potent (Fig 5A). Platelet-monocyte complex formation occurred readily upon stimulation with all *S*. *pneumoniae* serotypes tested (Fig 5B), platelet-lymphocyte complexes were not induced (Fig 5C). TLR2 and TLR4 were not directly involved in platelet-leukocyte complex formation as it was not induced by the TLR agonists LTA, Pam3CSK4 or LPS (shown for neutrophils, monocytes and lymphocytes in Fig 5D–5F).

### Wild-type mouse platelets respond to *S*. *pneumoniae D39* and *ΔcpsD39* in a similar manner as platelets from *Tlr2*^*-/-*^, *Tlr4*^*-/-*^, *Tlr2/4*^*-/-*^, *Tlr9*^*-/-*^ and *Myd88*^*-/-*^ mice

**Fig 6:**
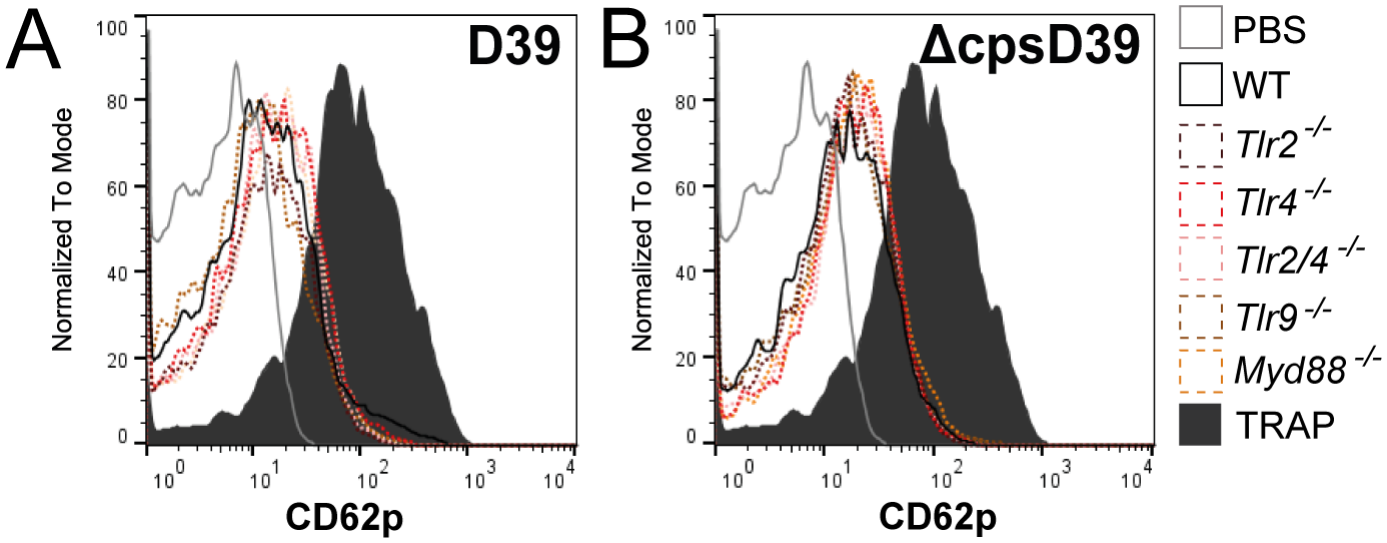
Wild-type mouse platelets respond to *S*. *pneumoniae* D39 and ΔcpsD39 in a similar manner as platelets from *Tlr2*^*-/-*^, *Tlr4*^*-/-*^, *Tlr2/4*^*-/-*^, *Tlr9*^*-/-*^ and *Myd88*^*-/-*^ mice. Mouse wild-type, *Tlr2*^*-/-*^, *Tlr4*^*-/-*^, *Tlr2/4*^*-/-*^, *Tlr9*^*-/-*^ and *Myd88*^*-/-*^ whole blood was stimulated with *S*. *pneumoniae* D39 (A) or ΔcpsD39 (B). Following 30 minutes of incubation platelets were stained and analysed by flow cytometry for surface expression of CD62p. N = 2–3 mice per group; histograms are representatives for the mice genotypes.

In order to test the contribution of TLR2 and 4 signalling in platelet responses to *S*. *pneumoniae* without the use of antibodies or synthetic agonists, we conducted similar whole blood stimulation experiments in mouse blood comparing wild-type platelets with platelets of *Tlr2*^*-/-*^, *Tlr4*^*-/-*^ and *Tlr2/4*^*-/-*^ strains using CD62p expression as readout for platelet activation. Recently, a functional role for platelet TLR9 was described [18]. We therefore included *Tlr9*^*-/-*^ mouse blood to investigate a possible role for TLR9 in this model. As a final control, we performed the stimulation experiments with blood obtained from *Myd88*^*-/-*^ mice, blocking downstream signalling of all TLR receptors except for TLR3 [17, 36]. *Tlr2*^*-/-*^, *Tlr4*^*-/-*^, *Tlr2/4*^*-/-*^, *Tlr9*^*-/-*^ and *Myd88*^*-/-*^ platelets all showed enhanced CD62p expression to a similar extent as wild-type platelets upon stimulation with *S*. *pneumoniae* D39 or ΔcpsD39, implicating that there is no role for TLR signalling in direct platelet response to *S*. *pneumoniae* (Fig 6A and 6B).

### Platelet MyD88 is not involved in host defence and response to *ΔcpsD39* in vivo

**Fig 7:**
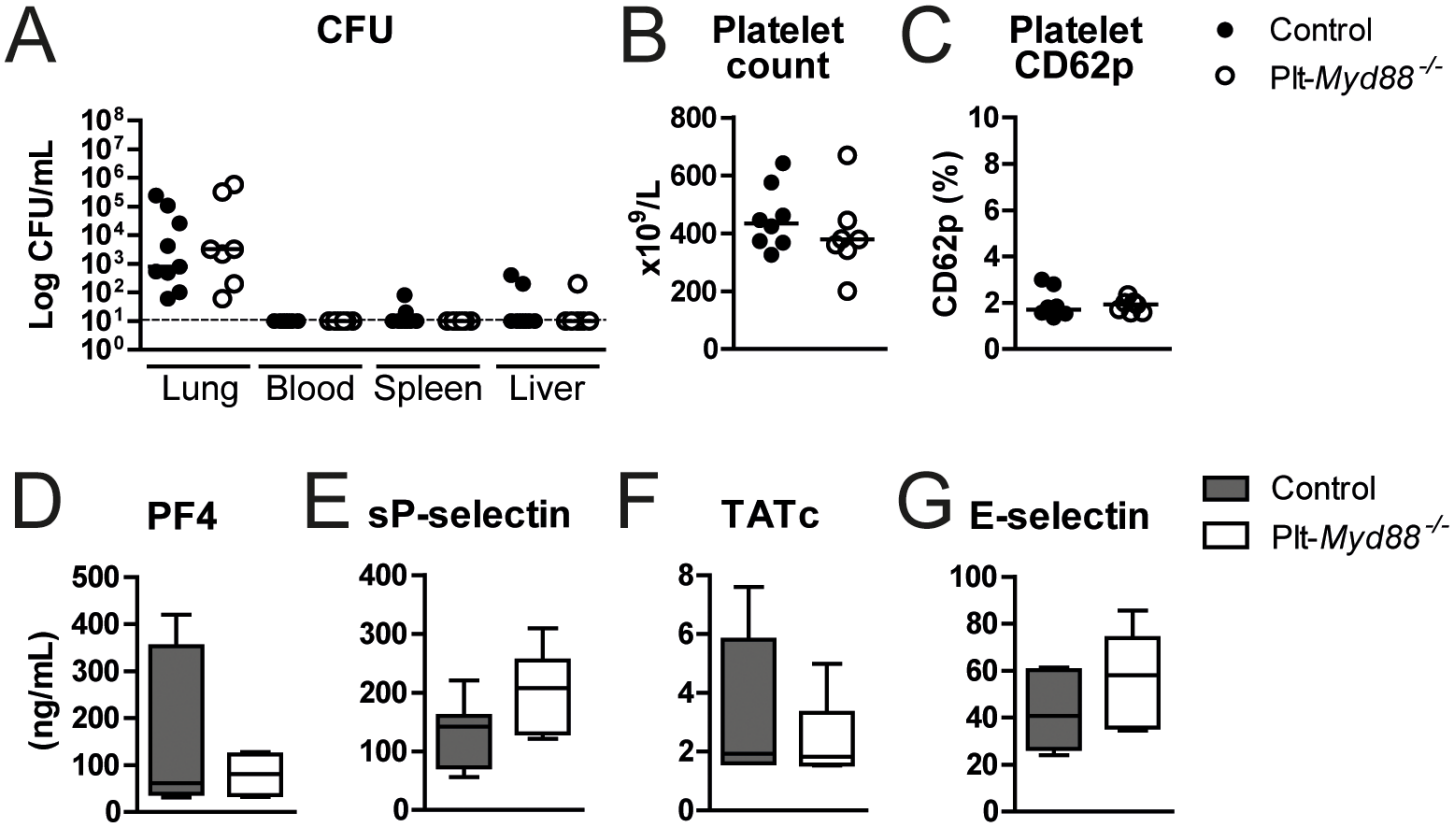
Platelet MyD88 is not involved in host defence to ΔcpsD39 *in vivo*. Control (closed dots, grey bars) and *Plt-Myd88*^*-/-*^ mice (open dots, white bars) were infected with *S*. *pneumoniae* ΔcpsD39 via the intranasal route and euthanized 16 hours thereafter. Bacterial counts were determined in lungs, blood, spleen and liver (A). Platelet counts (B) and platelet activation (CD62p; C) were determined by FACS analysis for CD61 and CD62p. PF4 (D), sP-selectin (E), TATc (F) and E-selectin (G) were measured in plasma using ELISA. Data are expressed as scatter dot plots or box- and whisker plots depicting the smallest observation, lower quartile, median, upper quartile and largest observation. N = 8 mice per group.

It is known that platelets especially exert proinflammatory and immune modulatory effects in the lungs [37]. To determine the impact of platelet specific TLR signalling during pneumonia *in vivo*, Plt-*Myd88*^*-/-*^ and littermate control mice were inoculated with 2 x 10^7^ CFU ΔcpsD39 via the airways. We chose to conduct these experiments with ΔcpsD39 which is cleared in an almost completely MyD88 dependent manner [38], and the strain that was the most potent inducer of platelet activation and platelet-leukocyte formation in our *in vitro* experiments. As control mice clear this unencapsulated *S*. *pneumoniae* strain within 24 hours, we therefore sacrificed the mice after 16 hours when bacterial loads are still present. No differences were detected in bacterial burdens in the lungs, blood, spleen or liver between control and Plt-*Myd88*^*-/-*^ mice (Fig 7A). Additionally, no differences were found in platelet counts (Fig 7B) or platelet activation measured by platelet surface CD62p (P-selectin) expression, PF4 and platelet and endothelial cell activation marker sP-selectin (Fig 7C–7E). (Activated) platelets are considered to play an essential role in coagulation by providing a phospholipid surface for the assembly of activated clotting factors [39]. To obtain insight in the role of MyD88 dependent platelet signalling in systemic coagulation activation during ΔcpsD39 pneumonia, we measured TATc levels in plasma of infected Plt-*Myd88*^*-/-*^ and control mice. No differences were detected between the groups (Fig 7F). Lastly, platelet MyD88 signalling had no influence on endothelial cell activation during ΔcpsD39 pneumonia as E-selectin levels did not significantly differ (Fig 7G).

**Fig 8:**
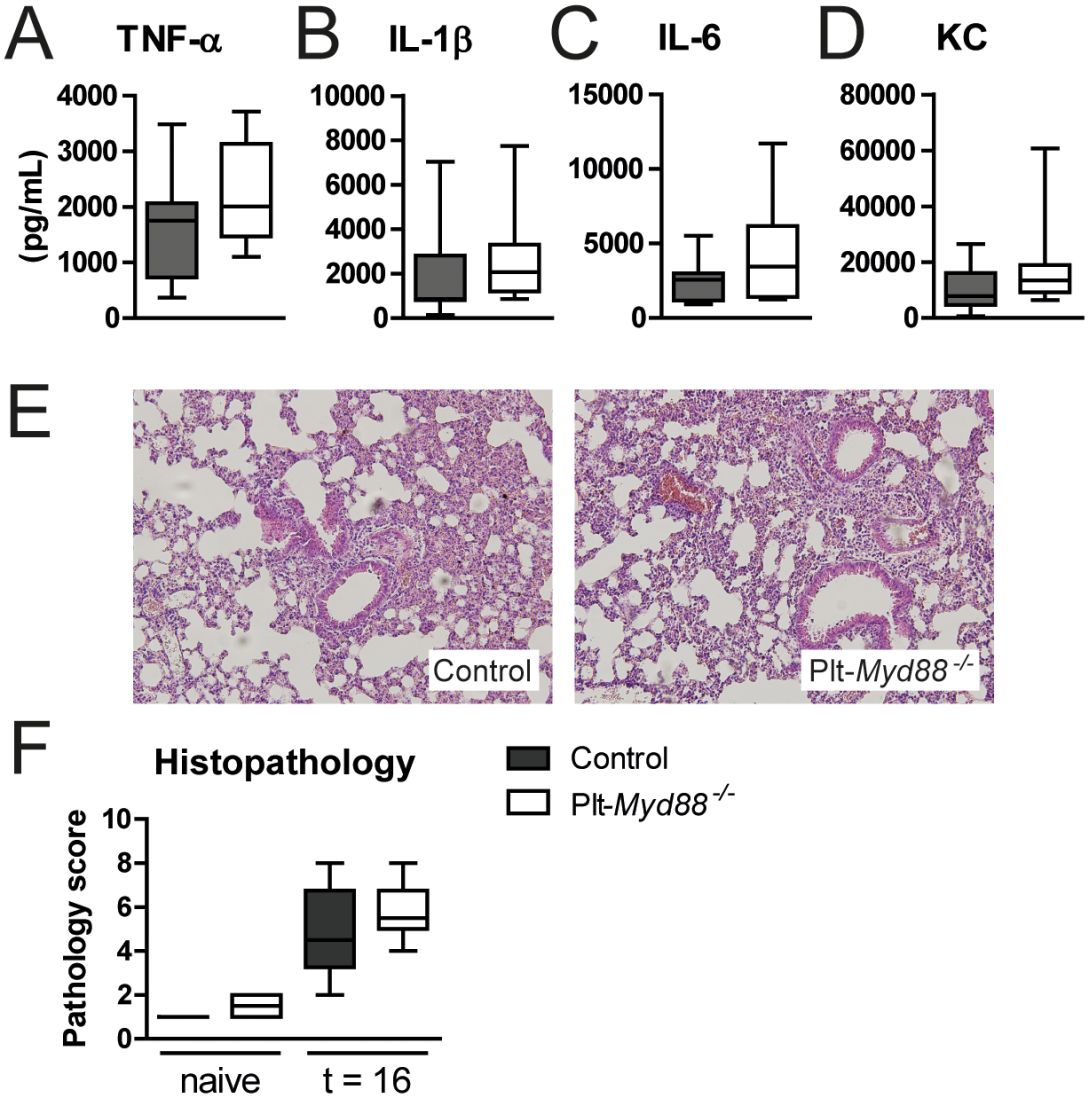
Platelet MyD88 is not involved in the inflammatory response to ΔcpsD39 *in vivo*. WT (gray bars) and *Plt-Myd88*^*-/-*^ mice (open bars) were infected with *S*. *pneumoniae* ΔcpsD39 via the intranasal route and euthanized 16 hours thereafter. Lung cytokine levels of TNF-α (A), IL-1β (B), IL-6 (C), and KC (D) were measured by ELISA. Lung histopathology was scored by an independent pathologist; representative microphotographs are shown in (E; 10x magnification) and pathology scores in (F). Data are depicted as are expressed as box- and whisker plots depicting the smallest observation, lower quartile, median, upper quartile and largest observation. N = 8 mice per group.

Platelets secrete inflammatory mediators upon activation like Platelet Factor 4 and RANTES [6] and platelets in complex with leukocytes can influence leukocyte effector function [4]. During ΔcpsD39 pneumonia however, cytokine production in the lungs did not differ between control and Plt-*Myd88*^*-/-*^ mice (Fig 8A–8D); plasma cytokine levels were below detection. Platelets have been both associated with enhanced histopathological damage during inflammatory challenges [40], and the protection of vascular integrity during inflammation [14, 15, 41]. However, no differences for inflammation parameters or infiltrated lung surface were found between Plt-*Myd88*^*-/-*^ and control mice, as reflected by the semi-quantitative scores of lung histopathology slides (Fig 8E and 8F). Additionally, no bleeding was found in the lungs of either Plt-*Myd88*^*-/-*^ or control mice.

## Discussion

*S*. *pneumoniae* represents a major health burden worldwide [42]. Recently, platelets have been implicated as major players in infection and immunity [6] and we have specifically shown this for *S*. *pneumoniae in vivo* [14]. Platelets are activated during sepsis, directly by an invading pathogen or indirect via injured endothelium and host coagulation activation [4]. In this paper we demonstrate that *S*. *pneumoniae* directly activates platelets in a TLR independent fashion. Platelet activation by all serotypes tested resulted in surface expression of CD62p and CD63 and platelet-leukocyte complex formation; ΔcpsD39 additionally induced platelet aggregation. In accordance, Plt-*Myd88*^*-/-*^ mice were unaffected during ΔcpsD39 pneumonia.

The pneumococcal capsule inhibits mucosal clearance, facilitates binding to the epithelial surface and inhibits complement- and phagocyte-mediated immunity [1]. Besides reduction of exposure to several antibodies, capsular polysaccharide was suggested to prevent interaction between Fcγ receptors to the Fc component of IgG bound to pneumococci [1, 43]. This could be why the only pneumococcal strain capable of inducing (FcyRII dependent) platelet aggregation was unencapsulated ΔcpsD39. Our results are in conflict with an earlier report showing that both encapsulated and unencapsulated *S*. *pneumoniae* induced platelet aggregation via TLR2 mediated signalling. The strains we tested however did not induce platelet aggregation unless in its mutated unencapsulated form (ΔcpsD39). ΔcpsD39 did not induce aggregation in a TLR2 dependent manner, as we could not inhibit the reaction by adding TLR2 blocking antibodies. In addition, direct TLR2 stimulation by TLR2 agonists LTA and Pam3CSK4 failed to induce platelet activation.

In contrast to the results found on platelet aggregation, we found that all strains of *S*. *pneumoniae* can induce platelet degranulation and complex formation and that the pneumococcal capsule only partly reduced this. In line with platelet aggregation, this was TLR independent, as blocking TLR-antibodies did not inhibit this and platelets from WT and *Tlr2*^*-/-*^, *Tlr4*^*-/-*^, *Tlr2/4*^*-/-*^, *Tlr9*^*-/-*^ and *Myd88*^*-/-*^ mice showed similar results. In contrast to platelet aggregation, this was not FcyRII dependent, as blocking FcγRII antibodies had no effect and mice (which lack FcγRII [44]) also show platelet degranulation and complex formation. It seems other (FcγRII independent) mechanisms are involved in platelet degranulation and complex formation, possibly GpIb, PECAM-1 or complement receptors [45–47].

Our results differ from a previously published paper by Keane et al., which found that encapsulated D39 (serotype 2) and TIGR4 (serotype 4) could induce platelet aggregation. Moreover, they found that anti-TLR2-antibodies could inhibit aggregation, whereas we found no role for TLR2 in interactions between platelets and *S*. *pneumoniae*. Several other papers have also reported functional roles for platelet TLRs *in vivo* [13, 19, 48–50], however controversy still surrounds the functionality of these receptors in *in vitro* assays. Stimulation of platelets with TLR2 or 4 ligands sometimes did [30, 51, 52] or did not [13, 34, 53] induce aggregation, did [34, 51] or did not [13, 19, 33] induce CD62p (P-selectin) expression, did [52, 54] or did not [33] induce Ca^2+^ mobilisation or thrombin generation [55]. We were unable to induce platelet activation by direct TLR2 agonists LTA and Pam3CSK4 or TLR4 agonist LPS, in a variety of functional assays. Possible differences between previous studies and ours (as well as differences between previous papers) remain difficult to clarify, but could well encompass technical issues such as culture method, amount and species of bacteria, quality of antibodies, PRP spinning protocols or platelet isolation methods, presence of plasma or different aggregometers.

Opposed to direct activation two groups found a priming effect on platelets of LPS alone [34], or in co-incubation with monocytes [53], whereafter platelets were ‘hyperexcitable’ and aggregated by addition of subthreshold levels of classic platelet agonists. Nevertheless, TLR agonists or encapsulated *S*. *pneumoniae* strains did not modulate the platelet response to subthreshold concentrations of TRAP in our hands.

The results presented have been generated in both murine and human blood. Although there are great similarities between mice and humans [56] differences obviously exist [57]. Therefore, caution must be taken when extrapolating results generated in mice. In the present study, *in vitro* data in human and murine blood showed similar results, with respect to the lack of involvement of TLR2 in activation of platelets by S. pneumoniae. Moreover, a recent study showed similar effects of platelets on host response in human sepsis patients as previously found in mice[58].

*In vitro* human experiments were performed using different donors. It has previously been reported that gender [59] and polymorphisms [60] can influence TLR expression and function. We observed similar results in 3 donors, but we cannot exclude effects of polymorphisms in this setting.

Platelets have been shown to be important in the host defence to *S*. *pneumoniae* pneumonia [14] and the unencapsulated serotype 2 strain D39 (ΔcpsD39) is cleared in a MyD88 dependent manner [38]. MyD88 dependent TLR signalling in platelets is not involved herein, as bacterial clearance was similar in Plt-*Myd88*^*-/-*^ and control mice during ΔcpsD39 pneumonia. In a gram negative pneumosepsis model using *K*. *pneumoniae*, we also observed no or minor contribution of platelet MyD88 dependent signalling. [61]. Moreover, platelet MyD88 deletion had no influence on platelet counts, platelet activation or coagulation activation.

Platelet TLR4 has been reported to modulate TNF-α production to bacterial lipopolysaccharide (LPS) [48]. Platelet activation during infection could additionally influence cytokine levels by release of cytokines from their own granules or by influencing leukocyte effector function [4, 6]. TNF-α and other cytokine levels were however similar in lungs of Plt-*Myd88*^*-/-*^ and control mice in our experiments. While platelets are additionally known to regulate lung architectural changes and vascular integrity during inflammation [40, 41], platelet activation via MyD88 dependent TLR signalling seems not involved as we found no histopathological differences between the groups in our pneumonia model. Differences with previous findings and the current could be explained by differences in type of bacteria used (gram positive or negative), dosis and model (inflammation vs. infection experiments).

The described activation patterns provide additional evidence that platelets function as circulatory sentinel cells in our immune system to detect and battle *S*. *pneumonia* as reported [62]. However, in this work we also show that *S*. *pneumoniae* apparently activates platelets by a mechanism that is independent of TLR signalling in platelets.

## Supporting Information

S1 Fig Testing of anti-TLR2 blocking antibodies in human whole blood. TLR2 blocking antibody’s were tested as described in material and methods. T2.5 was tested adequate and used in subsequent experiments.
(DOCX)

S2 Fig Prestimulation with S. pneumonia or TLR agonists fails to modulate human platelet aggregation in response to subthreshold concentrations of TRAP in presence of PBMCs. Prior to stimulation with this subthreshold TRAP concentration, PRP was incubated with S. pneumoniae D39, PAM3CSK4 or Lipopolysacharide (LPS) for 10 minutes in a stirring cuvette in the presence of Peripheral blood mononuclear cells (PBMCs).
(DOCX)
