# Supplementary material for: Toll-Like Receptor Signalling Is Not Involved in Platelet Response to Streptococcus pneumoniae In Vitro or In Vivo
Source: PLoS One. 2016 Jun 2;11(6):e0156977. doi: 10.1371/journal.pone.0156977 (PMC4890788; doi:10.1371/journal.pone.0156977)
Supplement: S1 Fig — TLR2 blocking antibody’s were tested as described in material and methods. T2.5 was tested adequate and used in subsequent experiments. (DOCX) [file pone.0156977.s001.docx]

**S1 Fig. Testing of anti-TLR2 blocking antibodies in human whole blood.** TLR2 blocking antibody’s were tested as described in material and methods. T2.5 was tested adequate and used in subsequent experiments.
