# Supplementary material for: Toll-Like Receptor Signalling Is Not Involved in Platelet Response to Streptococcus pneumoniae In Vitro or In Vivo
Source: PLoS One. 2016 Jun 2;11(6):e0156977. doi: 10.1371/journal.pone.0156977 (PMC4890788; doi:10.1371/journal.pone.0156977)
Supplement: S2 Fig — Prior to stimulation with this subthreshold TRAP concentration, PRP was incubated with S. pneumoniae D39, PAM3CSK4 or Lipopolysacharide (LPS) for 10 minutes in a stirring cuvette in the presence of Peripheral blood mononuclear cells (PBMCs). (DOCX) [file pone.0156977.s002.docx]

**

**

**S2 Fig. Prestimulation with S. pneumonia or TLR agonists fails to modulate human platelet aggregation in response to subthreshold concentrations of TRAP in presence of PBMCs.** Prior to stimulation with this subthreshold TRAP concentration, PRP was incubated with S. pneumoniae D39, PAM3CSK4 or Lipopolysacharide (LPS) for 10 minutes in a stirring cuvette in the presence of Peripheral blood mononuclear cells (PBMCs).
